# *BDKRB2* +9/−9 Polymorphism Is Associated with Higher Risk for Diabetes Mellitus in the Brazilian General Population

**DOI:** 10.1155/2012/480251

**Published:** 2012-11-29

**Authors:** Rafael de Oliveira Alvim, Paulo C. J. L. Santos, Raimundo M. Nascimento, George L. L. M. Coelho, José G. Mill, José E. Krieger, Alexandre C. Pereira

**Affiliations:** ^1^Laboratory of Genetics and Molecular Cardiology, Heart Institute (InCor), University of Sao Paulo Medical School, Avenida Dr. Enéas de Carvalho Aguiar, 44 Cerqueira César, 05403-000 São Paulo, SP, Brazil; ^2^Department of Medicine, Ouro Preto Federal University, Ouro Preto, MG, Brazil; ^3^Department of Physiology, Espirito Santo Federal University, Vitória, ES, Brazil

## Abstract

Some mechanisms have been proposed to explain the role of bradykinin on glucose homeostasis and some studies reported that the *BDKRB2* +9/−9 polymorphism was associated to the transcriptional activity of the receptor. In this scenario, the main aim of this study was to evaluate the association of the *BDKRB2* +9/−9 polymorphism with diabetes mellitus risk in the Brazilian general population. This study included 1,032 subjects of the general urban population. Anthropometrical, blood pressure, biochemical, and genotype analyses for the *BDKRB2* +9/−9 bp insertion/deletion polymorphism were performed. Individuals carrying +9/+9 or +9/−9 genotypes had higher glucose values (84.5 mg/dL versus 80.6 mg/dL, resp.) and higher frequency of diabetes mellitus (7.6% versus 3.6%, resp.) compared to individuals carrying −9/−9, adjusting for age and gender. In addition, higher diabetes mellitus risk was associated to presence of the +9/+9 or +9/−9 genotypes (OR = 1.91; 95% CI = 1.09–4.19; *P* = 0.03). Our data suggest that the *BDKRB2* +9/-9 polymorphism may act as a genetic modulator of glucose homeostasis. It was previously associated to insulin sensitivity, glucose uptake, and insulin secretion, and, in this study, data suggest that the polymorphism may increase susceptibility to chronic metabolic conditions such as diabetes in the Brazilian population.

## 1. Introduction

Bradykinin (BK) is a nonapeptide formed by the action of a serine protease called kallikrein. The kallikrein produces kinins which are largely released into interstitial fluid, blood, and glandular tissue, in particular by the pancreas. Several studies have demonstrated the role of BK in the modulation of important physiological effects such as inflammation, vascular permeability, hypotension, edema, smooth muscle contraction, and glucose homeostasis, and most of these actions are mediated by the B2 receptor (B2R) [1, 2].

 Recently, some mechanisms have been proposed to explain the role of BK on glucose homeostasis. Studies have reported that BK enhances the tyrosine phosphorylation of IRS1 and thus improves the binding affinity of IRS1 with the P85 regulatory subunit of PI3K, which increases the translocation of GLUT4 to the plasma membrane [3]. In addition, the activation of endothelial nitric oxide synthase (eNOS) by BK results in improved blood flow thereby increasing glucose supply to peripheral tissues [4].

 B1 or B2 receptors mediate the effects of BK. It has been accepted that almost all of the physiologically significant effects of BK, including the metabolic ones, are exerted by activation of the B2R [5]. Based on this information, several studies have focused on insertion/deletion polymorphism in the exon 1 of the BK type 2 receptor gene (*BDKRB2*). The presence (+9 bp) rather than the absence (−9 bp) of a 9-base pair sequence was associated to lower gene transcriptional activity [6, 7]. The main aim of this study was to evaluate the association of the *BDKRB2* +9/−9 polymorphism with diabetes mellitus risk in the Brazilian general population.

## 2. Methods

### 2.1. Study Population

 This study included 1,032 subjects of the general urban population selected from the Hearts of Brazil Project (HBP). The design was a transversal and multicenter observational cohort study. The universe of the HBP consisted in the set of inhabitants of Brazilian urban centers with more than 100,000 inhabitants. The HBP sample plan was calculated as 2,500 interviews, distributed in 72 cities from the 5 regions of the country proportionally to the number of inhabitants, per gender and age range, based on data from IBGE (Brazilian Census). In the selected cities, the “households” constituted the second-stage units, with one interview per household. Subjects were separated in self-declared “racial/color” subgroups, according to Brazilian Census, as White, Intermediate (meaning Brown, *Pardo* in Portuguese), or Black [8–10]. The study protocol was approved by the involved Institutional Ethics Committees and written informed consent was obtained from all participants prior to entering the study. 

### 2.2. Anthropometrical, Blood Pressure, and Biochemical Data

 Weight, height, and waist were measured according to a standard protocol. Body mass index (BMI) was calculated and obesity defined as BMI ≥ 30 kg/m^2^. Blood pressure was measured in the sitting position with the use of a standard mercury sphygmomanometer on the left arm after 5 minutes' rest. The first and fifth phases of Korotkoff sounds were used for systolic (SBP) and diastolic blood pressure (DBP), respectively. Hypertension was defined as mean SBP ≥ 140 mmHg and/or DBP ≥ 90 mmHg or use of anti-hypertension drugs [11]. Fasting glucose, triglycerides (TG), and total cholesterol (TC) were assayed by technology point-of-care (Roche Diagnostics, Accu-Check). Diabetes was defined as fasting glucose ≥ 126 mg/dL or hypoglycemic drugs use according to physician prescription (previous diabetes) [12].

### 2.3. Genotyping

 Genomic DNA was extracted from peripheral blood leukocytes following a salting-out method. Genotypes for the *BDKRB2 *+9/−9 bp insertion/deletion polymorphism was performed by polymerase chain reaction followed by restriction fragment length polymorphism (PCR-RFLP) as previously described [13]. Quality control for these assays was assessed by randomly selecting 10% samples to be re-genotyped by two independent technicians. 

### 2.4. Statistical Analyses

 Statistical analyses were carried out using the SPSS 19.0 software (Chicago, IL, USA), with the level of significance set at *P* ≤ 0.05. Categorical variables were presented as percentage, whereas continuous variables were presented as mean ± standard deviation. Differences in the categorical and continuous parameters according to genotypes for the *BDKRB2* polymorphism were tested by chi-square test and Student's *t*-test, respectively. Logistic regression analyses were performed to evaluate the association of the *BDKRB2* polymorphism with diabetes mellitus plus adjustment for covariates. 

## 3. Results 

 Demographic and clinical data of the studied population samples are shown in Table 1. Genotypic frequencies were 26.8% for +9/+9; 53.4% for +9/−9; 19.8% for −9/−9, and frequency of deletion allele was of 47%. Genotypic distribution in the overall sample was in accordance with the Hardy-Weinberg equilibrium (*P* = 0.46, *χ* ^2^ = 0.54).

 Differences in age, gender, race/color, BMI, obesity, abdominal circumference, SBP, DBP, hypertension, TG, and TC according to genotype groups were not observed (+9/+9 plus +9/−9 versus −9/−9). However, individuals carrying +9/+9 or +9/−9 genotypes had higher glucose values (84.5 mg/dL versus 80.6 mg/dL; *P* = 0.04, resp.) and higher frequency of diabetes mellitus (7.6% versus 3.6%, resp.) compared to individuals carrying −9/−9, adjusting for age, gender, and BMI (Table 1). In addition, higher diabetes mellitus risk was associated to presence of the +9/+9 or +9/−9 genotypes (OR = 1.91; 95% CI = 1.09–4.19; *P* = 0.03) (Table 1).

## 4. Discussion

 The main finding of this study was that the *BDKRB2* +9/−9 polymorphism is associated with fasting glucose values and with diabetes mellitus risk in the Brazilian population. These genetic associations, although exploratory regarding casual relations, may have a role in the generation of hypothesis to be tested in more controlled studies. 

 In recent years, numerous studies have demonstrated the important biological role of BK in the modulation of various physiological conditions mediated through its binding to B1 and B2 receptors [1, 2]. In addition, a recent study reported that the B2 receptor mediates the interaction of BK with pathways related to glucose homeostasis [5].

 Some investigations confirmed the key role of BK on insulin sensitivity [14], glucose uptake [15], and insulin secretion by beta cells [16]. Iozzo et al., studying obese and lean individuals, demonstrated that intra-arterial infusion of BK in the left leg resulted in an increase in glucose uptake restricted to lean subjects [17]. Corroborating the above results, Uehara et al. demonstrated that infusion of BK increased glucose uptake into dog peripheral tissues and that infusion of a BK antagonist abolished this effect on insulin sensitivity [18]. 

Finally, Yang and Hsu, studying rat pancreas, showed that the infusion of BK increased the insulin release by beta cells. Furthermore, the BK optimized insulin release stimulated by glucose [16]. In this scenario and knowing that the presence (+9 bp) rather than the absence (−9 bp) of a 9-base pair sequence was associated to lower gene transcriptional activity [6, 7], our results suggest a role of BK on glucose homeostasis represented by increased risk for diabetes mellitus.

 Considering the pivotal role of B2 receptor in the physiological actions of BK, studies evaluating this genetic variant with metabolic phenotypes are still scarce in the literature. Our findings are supported by functional studies in the literature [14–17]. Moreover, previous studies demonstrated the role of the B2 receptor in the metabolic actions of BK [5] and association of *BDKRB2* +9 allele with lower gene transcriptional activity [7]. Finally, our data may suggest that the reduced transcriptional activity of the B2 receptor presented by +9 allele carriers is associated to lower BK activity, reduced glucose uptake [15], and insulin resistance [14]. Thus, it could generate an unfavorable glucose profile and consequent increased risk for developing diabetes.

 There are some limitations in our study. First, we were not able to specifically determine the molecular alteration responsible for the observed association. Nonetheless, in the described scenario, our data plus literature data are well grounded and provide evidence that the *BDKRB2* gene may constitute not only a link between biomechanical transduction and diabetes condition, but may also be a fundamental gateway for both epidemiological and mechanistic studies involving diabetes, hypertension, and renin-angiotensin system hyperactivity. Second, our examination of glucose has its limitations against the gold standard. However, the classification of the diabetes condition was made according to criteria recommended [12]. Finally, it is not possible to completely exclude the interaction between the use of other genetic markers, ethnicity, concomitant drugs, and other covariates on our findings [19, 20].

 In conclusion, our data suggest that the *BDKRB2* +9/−9 polymorphism may act as a genetic modulator of glucose homeostasis. It was previously associated to insulin sensitivity [14], glucose uptake [15], and insulin secretion by beta cells [16] and, in this study, data suggest that the polymorphism may increase susceptibility to chronic metabolic conditions such as diabetes in the Brazilian population. 

## Figures and Tables

**Table 1:** Demographic, clinical, and biochemical data according to genotypes for the *BDKRB2 *polymorphism.

Subject Characteristics	+9/+9 /+9/−9		−9/−9		*P* value
*n*, (%)	845 (80.2%)		208 (19.8%)		
Gender, male	386 (45.7%)		99 (47.6%)		0.62
Ethnicity, *n* (%)					
African descent	81 (9.6%)		17 (8.2%)		0.63
Caucasian descent	493 (58.3%)		126 (60.6%)		
Mullato	256 (30.3%)		59 (28.4%)		
Others	15 (1.8%)		6 (2.9%)		
Diabetes, *n* (%)	64 (7.6%)		8 (3.6%)		**0.03**
Obesity, *n* (%)	183 (24.4%)		41 (21.6%)		0.41
Age, years	42.7 ± 14.1		44.4 ± 15.2		0.12
BMl*, kg/m^2^	27.0 ± 5.1		26.4 ± 4.8		0.19
Abdominal Circumference, cm	90.5 ± 13.6		90.4 ± 13.3		0.90
SBP^†^, mmHg	128.6 ± 23.4		126.2 ± 22.1		0.13
DBP^†^, mmHg	80.1 ± 14.3		78.4 ± 12.4		0.07
Glucose^†^, mg/dL	84.5 ± 28.3		80.6 ± 21.9		**0.04**
Triglycerides^†^, mg/dL	154.2 ± 86.4		146.9 ± 71.2		0.33
Total cholesterol^†^, mg/dL	193.4 ± 35.4		187.7 ± 33.2		0.13

			OR [95% CI], *P* value		
			Diabetes Mellitus		
		Unadjusted		Adjusted^†^	

Genetic Models of Inheritance +9/+9/+9/−9 genotype group		2.05 [0.97–4.34]		1.91 [1.09–4.19]	
		0.06		0.03	

*Adjusted for gender and age.

^†^Adjusted for gender, age, and BMI.

BMI: body mass index; SBP: systolic blood pressure; DBP: diastolic blood pressure.

“Race/color” was classified according to Brazilian Census as White, Intermediate (meaning Brown, *Pardo *in Portuguese), or Black.
